# Positive association between serum uric acid and metabolic dysfunction-associated steatotic liver disease: insights from a Japanese health checkup cohort

**DOI:** 10.1186/s12902-026-02174-5

**Published:** 2026-01-30

**Authors:** Mari Arikawa, Mizue Saita, Hirohide Yokokawa, Yuichi Takahashi, Hiroshi Fukuda, Toshio Naito

**Affiliations:** Department of General Medicine, Juntendo University Faculty of Medicine, 2-1-1 Hongo, Bunkyo-ku, Tokyo, 113-8421 Japan

**Keywords:** Metabolic dysfunction-associated steatotic liver disease, Serum uric acid, Metabolic diseases, Non-alcoholic fatty liver disease, Preventive medicine, Fatty liver

## Abstract

**Background:**

In 2023, non-alcoholic fatty liver disease (NAFLD) was defined as metabolic dysfunction-associated steatotic liver disease (MASLD) to avoid stigmatizing terminology. Although recent studies have suggested an association between MASLD and serum uric acid (SUA), the evidence remains limited. In this study, we aimed to assess the association between MASLD and SUA in Japanese participants.

**Methods:**

This cross-sectional study included 3,264 eligible participants (1,917 men and 1,347 women) who underwent voluntary health checkups at Juntendo University Hospital, Tokyo, Japan, between January 2021 and December 2023. Data on basic characteristics, anthropometric indices, blood pressure, glycemic and lipid markers, SUA levels, and medical histories were collected. Steatosis was assessed by computed tomography or ultrasound. MASLD was defined as imaging-confirmed steatosis plus at least one metabolic dysfunction criterion. The participants were stratified into sex-specific SUA quartiles. Associations between MASLD and SUA quartiles were evaluated by multivariable logistic and Poisson regression, adjusted for age, alcohol consumption, smoking, estimated glomerular filtration rate, urate-lowering medication use, and body mass index. The results are reported as adjusted odds ratios (AORs) or prevalence ratios (PRs) with 95% confidence intervals (CIs).

**Results:**

Higher SUA levels were significantly associated with MASLD prevalence. After adjustment for confounders, the association remained significant. Compared with the lowest quartile, the AORs (95% CI) for MASLD in men were: Q2, 1.45 (1.08–1.97, *p* < 0.01); Q3, 1.43 (1.04–1.95, *p* < 0.01); and Q4, 2.00 (1.45–2.76, *p* < 0.01). In women, the AORs were: Q2, 1.45 (0.90–2.35, *p* = 0.13); Q3, 2.21 (1.39–3.53, *p* < 0.01); and Q4, 3.52 (2.22–5.56, *p* < 0.01). SUA that was treated as a continuous variable showed a clear dose–response association in both sexes. Sensitivity analyses using imaging-defined steatosis yielded consistent results.

**Conclusions:**

SUA levels were positively associated with MASLD in both men and women, with women showing elevated MASLD risk even at SUA levels below the conventional hyperuricemia threshold. SUA might serve as a clinically relevant metabolic indicator; consideration of sex-specific metabolic risk might improve MASLD risk assessment. Further research on clarifying causal pathways is warranted.

**Clinical trial number:**

Not applicable.

**Supplementary Information:**

The online version contains supplementary material available at 10.1186/s12902-026-02174-5.

## Background

Non-alcoholic fatty liver disease (NAFLD), a condition characterized by intrahepatic fat accumulation not explained by excessive alcohol intake, is strongly linked to metabolic syndrome and insulin resistance [1–3]. The global prevalence is approximately 20–40%; epidemiological studies in Japan reported prevalence rates of approximately 30% [4–6].

In 2023, NAFLD was redefined as metabolic dysfunction–associated steatotic liver disease (MASLD) to emphasize its cardiometabolic basis and reduce stigmatizing terminology [7]. MASLD requires evidence of hepatic steatosis plus at least one metabolic abnormality such as obesity, glucose intolerance, hypertension, or dyslipidemia. With increasing visceral obesity, MASLD is expected to become more common and recognized as a risk factor for cardiovascular and cerebrovascular diseases [8].

Hyperuricemia is a prevalent metabolic disorder associated with cardiovascular disease, type 2 diabetes mellitus (T2DM), and hypertension [9, 10]. Its relationship with NAFLD has been well documented, potentially mediated by hyperinsulinemia and increased xanthine oxidoreductase activity [11–14]. After adopting the MASLD definition, several studies have reported associations between SUA and MASLD [15–18]. However, evidence from Japanese general populations remains limited.

Therefore, in this study, we aimed to evaluate the association between SUA and MASLD in a large sample of Japanese adults and explore potential sex-specific differences using detailed biochemical, imaging, and lifestyle data.

## Methods

This cross-sectional study included 6,906 Japanese individuals who underwent health checkups at Juntendo University Hospital, a 1,051-bed tertiary care university hospital located in central Tokyo, Japan, between January 2021 and December 2023. Participants with complete data on physical measurements, blood test results, and abdominal imaging by either computed tomography (CT) scan or ultrasonography were considered eligible for inclusion.

A total of 3,619 individuals were excluded because they underwent health checkups more than twice during the study period, resulting in duplicate cases. An additional 23 individuals were excluded due to missing data. Ultimately, data of 3,264 participants were included in the final analysis.

The Ethics Committee of Juntendo University reviewed and approved the research protocol using retrospective data (approval number: E23-0431). Written informed consent was obtained from all participants at the time of their health checkup.

### Variables

MASLD was diagnosed according to the criteria jointly established in June 2023 by the European Association for the Study of the Liver, American Association for the Study of Liver Diseases, and Latin American Association for the Study of Liver Diseases. MASLD was diagnosed when hepatic steatosis was confirmed by imaging or liver biopsy in combination with at least one cardiometabolic risk factor, including obesity, T2DM, hypertension, hypertriglyceridemia, or low High-density lipoprotein cholesterol (HDL-C) levels [7].

Height, body weight, body mass index (BMI), and waist circumference (WC) were measured while the participants stood in test clothing. BMI was calculated as body weight (kg) divided by height squared (m^2^). Systolic blood pressure (SBP) and diastolic blood pressure (DBP) were measured from the upper arm using an automatic sphygmomanometer after the participants were seated for at least 5 min.

Fasting blood samples were collected and immediately submitted for biochemical analysis. The following parameters were measured: total cholesterol (TC, mg/dL), HDL-C (mg/dL), low-density lipoprotein cholesterol (LDL-C, mg/dL), and triglycerides (TGs, mg/dL). LDL-C levels were determined using the direct measurement method. Fasting plasma glucose (FPG) and glycosylated hemoglobin (HbA1c; National Glycohemoglobin Standardization Program) were also assessed. HbA1c levels were determined by high-performance liquid chromatography using an automated analyzer. Serum uric acid (SUA, mg/dL), aspartate aminotransferase (AST, U/L), and alanine aminotransferase (ALT, U/L) were also measured. Estimated glomerular filtration rate (eGFR) was calculated using the Japanese GFR equation: eGFR (mL/min/1.73 m^2^) = 194 × Cr^− 1.094^ × age^− 0.287^ × (0.739 for women) [19]. As part of the blood collection, a specific contamination-preventing blood collection tube was used.

Fatty liver was assessed by CT scanning using a 320-row CT system (Aquilion ONE/GENESIS Edition; Canon Medical Systems, Tokyo, Japan). Imaging was performed at the umbilical level with participants in the supine position during late expiration. Fatty liver was determined when the liver-to-spleen CT density ratio (L/S ratio) was less than 0.9.

Ultrasonography was performed using a TOSHIBA Aplio 500 system. The diagnostic criteria for fatty liver included hyperintense liver, hepatorenal or hepatosplenic contrast, deep attenuation enhancement, and obscuration of intrahepatic vessels. The revised edition of the Abdominal Ultrasound Screening and Assessment Manual (2021) was used as a reference. All imaging examinations were performed by experienced clinical laboratory technologists.

The participants completed a self-administered questionnaire assessing lifestyle characteristics based on Breslow’s seven health practices [20]. The questions were about alcohol consumption, smoking status, sleep duration, exercise frequency per week, frequency of breakfast, snacking between meals, and weight management. Medical history of comorbidities such as hypertension, diabetes mellitus (DM), dyslipidemia, hyperuricemia, cardiovascular disease, and cerebrovascular disease was also collected from the questionnaire. The participants who indicated having any of these conditions were recorded as positive for the respective comorbidity.

### Statistical analysis

Demographic and biochemical characteristics are summarized using appropriate descriptive statistics. Continuous variables are expressed as mean and standard deviation (SD), while categorical variables are presented as prevalence frequency and percentage. Group comparisons of categorical variables were conducted using the chi-square test. Continuous variables that followed a normal distribution were analyzed using the t-test or analysis of variance (ANOVA) and presented as mean ± SD.

To assess the association between SUA levels and the prevalence of MASLD, clinical and biochemical characteristics were compared across sex-specific SUA quartiles. Quartiles were defined as follows: men, Q1 < 5.2, 5.2 ≤ Q2 < 6.0, 6.0 ≤ Q3 < 6.7, 6.7 ≤ Q4 (mg/dL); women, Q1 < 4.1, 4.1 ≤ Q2 < 4.7, 4.7 ≤ Q3 < 5.4, 5.4 ≤ Q4 (mg/dL). SUA was also analyzed as a continuous variable. Analyses were stratified by sex. Group values were compared using one-way ANOVA; statistical significance was assessed using the chi-square test.

Associations between MASLD and SUA were evaluated using multivariable logistic regression analysis to calculate adjusted odds ratios (AORs) and 95% confidence intervals (CIs). Analyses were performed for unadjusted and adjusted models. Model 1 was adjusted for age. Model 2 was adjusted for age, alcohol consumption (non-daily drinker), and smoking status (non-smoker), eGFR, and use of urate-lowering medications. Model 3 was further adjusted for BMI. These covariates were selected based on their known influence on SUA levels [9] and potential associations with MASLD. In contrast, lipid and glycemic markers were not included as covariates, because they overlap with MASLD diagnostic components and might introduce overadjustment. Multivariable logistic regression was performed separately in men and women, given previous reports indicating sex differences in uric acid levels [21]. A two-sided *P* < 0.05 was considered statistically significant.

Since the prevalence of MASLD in this cohort exceeded 40%, AORs from logistic regression might substantially overestimate relative differences. Therefore, PRs were additionally estimated using Poisson regression with a log link and robust variance. Poisson models were constructed in parallel with the logistic models (Model, 1–3), using MASLD as the dependent variable and SUA quartiles as the main exposure. Sex-stratified analyses were also performed for the Poisson models.

As a sensitivity analysis to minimize potential circularity arising from the metabolic components of the MASLD definition, we additionally evaluated the association between SUA and imaging-defined hepatic steatosis (fatty liver vs. non-fatty liver), independent of metabolic criteria. Logistic and Poisson regression models identical to the main analysis were applied; a separate analysis restricted to ultrasound-only assessments was also performed.

To evaluate the robustness of the observed associations, we performed several additional analyses. First, SUA–MASLD associations were recalculated in imaging-based subgroups. Since the number of CT-defined steatosis cases was too small to permit stable multivariable estimation, subgroup analyses were performed using ultrasound-defined steatosis only. Second, E-values were computed for key fully adjusted estimates (Model 3) to quantify the minimum strength of unmeasured confounding required for explaining away the observed associations. Finally, SUA was modeled as a continuous variable to assess dose–response patterns. Restricted cubic spline (RCS) models with four knots were additionally applied to evaluate potential non-linear associations between SUA and MASLD, stratified by sex. Non-linearity was assessed using Wald tests for the spline terms.

All primary statistical analyses were performed using the Statistical Package for the Social Sciences, version 29 (SPSS Inc., Chicago, IL, USA). The RCS analyses were conducted using R (version 4.5.2) with the rms package.

## Results

Table 1 presents the demographic and biochemical characteristics of the 3,264 participants, stratified by sex. The mean (SD) age was 61.0 (13.1) years in men and 61.4 (12.8) years in women. Treatment of hyperuricemia was reported in 308 men (16.1%) and 12 women (0.9%). Among all participants, 1,349 (41.3%; 952 men and 397 women) fulfilled the diagnostic criteria for MASLD.

**Table 1:** Sex-specific characteristics of the participants

	Mean (SD) or *N* (%)		*P*
	Men (*n* = 1,917)	Women (*n* = 1,347)	
Age (years)	61.0 (13.1)	61.4 (12.8)	0.30
Anthropometric measurements			
Body mass index (kg/m^2^)	24.4 (3.4)	22.0 (3.8)	< 0.01
Waist circumference (cm)	86.1 (9.4)	78.4 (10.3)	< 0.01
Healthy lifestyle characteristics			
Alcohol consumption (daily drinker)	656 (34.2)	217 (16.1)	< 0.01
Smoking behavior (current smoker)	243 (12.7)	68 (5.0)	< 0.01
Blood pressure measurements			
Systolic blood pressure (mmHg)	124.6 (14.5)	121.3 (17.1)	< 0.01
Diastolic blood pressure (mmHg)	75.0 (10.4)	70.7 (11.4)	< 0.01
< 140 (SBP) and 90 (DBP) (mmHg)	1556 (81.1)	1116 (82.9)	0.21
Diabetes medication (yes)	263 (13.7)	64 (4.8)	< 0.01
Fasting blood glucose (mg/dL)	106.1 (19.6)	98.3 (15.2)	< 0.01
Hemoglobin A1c (%)	5.9 (0.7)	5.8 (0.5)	< 0.01
Dyslipidemia medication (yes)	541(28.2)	284 (21.1)	< 0.01
Total cholesterol (mg/dL)	198.9 (35.3)	220.0 (35.9)	< 0.01
High-density lipoprotein cholesterol (mg/dL)	55.6 (13.9)	69.7 (16.8)	< 0.01
Low-density lipoprotein cholesterol (mg/dL)	112.8 (30.2)	120.7 (30.6)	< 0.01
Triglycerides (mg/dL)	122.7 (88.1)	91.6 (53.5)	< 0.01
Hyperuricemia medication (yes)	308 (16.1)	12 (0.9)	< 0.01
Uric acid (mg/dL)	6.0 (1.2)	4.8 (1.1)	< 0.01
Organ damage/cardiovascular disease			
Cardiovascular disease	149 (7.8)	49 (3.6)	< 0.01
Cerebrovascular disease	40 (2.1)	37 (2.7)	0.23
CKD (eGFR < 60 mL/min/1.73 m^2^)	403 (21.0)	191 (14.2)	< 0.01
eGFR (mL/min/1.73 m^2^)	71.5 (15.4)	75.0 (15.2)	< 0.01
Liver function			
Aspartate aminotransferase (U/L)	24.8 (10.8)	22.8 (8.2)	< 0.01
Alanine aminotransferase (U/L)	26.6 (18.8)	19.5 (12.8)	< 0.01

SD, standard deviation; N, number; SBP, systolic blood pressure; DBP, diastolic blood pressure; CKD, chronic kidney disease; eGFR, estimated glomerular filtration rate

Table 2 lists the demographic and biochemical characteristics of the participants with and without MASLD by sex. The MASLD group demonstrated significantly higher BMI, WC, SBP, DBP, FPG, HbA1c, TC, LDL-C, TGs, AST, and ALT levels, along with significantly lower HDL-C levels. Notably, SUA levels were also significantly higher in the participants with MASLD compared with those without it.

**Table 2:** Basic characteristics of MASLD and non-MASLD participants by sex

	MASLD (*n* = 1,349)					non-MASLD (*n* = 1,915)				
	Mean (SD) or *N* (%)					Mean (SD) or *N* (%)				
	Men (*n* = 952)		Women (*n* = 397)			Men (*n* = 965)		Women (*n* = 950)		
Age (years)	60.7 (11.8)		63.4 (11.7)		< 0.01	61.3 (14.3)		60.6 (13.1)		0.31
Anthropometric measurements										
Body mass index (kg/m^2^)	26.0 (3.5)		25.1 (4.2)		< 0.01	22.9 (2.5)		20.7 (2.7)		< 0.01
Waist circumference (cm)	90.3 (9.2)		86.3 (9.9)		< 0.01	82.0 (7.7)		75.1 (8.5)		< 0.01
Healthy lifestyle characteristics										
Alcohol consumption (daily drinker)	311 (32.7)		59 (14.9)		< 0.01	345 (35.7)		158 (16.6)		< 0.01
Smoking behavior (current smoker)	141 (14.8)		24 (6.0)		< 0.01	102 (10.6)		44 (4.6)		< 0.01
Blood pressure measurements										
Systolic blood pressure (mmHg)	126.3 (14.3)		127.0 (16.5)		0.49	123.0 (14.5)		118.9 (16.8)		< 0.01
Diastolic blood pressure (mmHg)	76.6 (10.4)		73.5 (11.9)		< 0.01	73.3 (10.2)		69.5 (10.9)		< 0.01
< 140 (SBP) and 90 (DBP) (mmHg)	743 (78.0)		301 (75.8)		< 0.01	812 (84.1)		815 (85.8)		0.38
Diabetes medication (yes)	176 (18.5)		44 (11.1)		< 0.01	87 (9.0)		20 (2.1)		< 0.01
Fasting blood glucose (mg/dL)	111.0 (21.7)		105.3 (19.2)		< 0.01	101.4 (15.8)		95.3 (11.9)		< 0.01
Hemoglobin A1c (%)	6.1 (0.7)		6.0 (0.6)		0.26	5.7 (0.5)		5.7 (0.4)		< 0.01
Dyslipidemia medication (yes)	306 (32.1)		128 (32.2)		0.97	235 (24.3)		156 (16.4)		< 0.01
Total cholesterol (mg/dL)	201.9 (37.4)		219.6 (37.8)		< 0.01	195.9 (32.8)		220.1 (35.0)		< 0.01
High-density lipoprotein cholesterol (mg/dL)	51.8 (13.0)		61.1 (14.3)		< 0.01	59.4 (13.7)		73.3 (16.5)		< 0.01
Low-density lipoprotein cholesterol (mg/dL)	117.4 (32.1)		127.1 (33.1)		< 0.01	108.3 (27.3)		118.0 (29.0)		< 0.01
Triglycerides (mg/dL)	147.7 (102.1)		124.1 (70.8)		< 0.01	97.9 (62.5)		78.0 (36.6)		< 0.01
Hyperuricemia medication (yes)	190 (20.0)		3 (0.8)		< 0.01	118 (12.2)		9 (0.9)		< 0.01
Uric acid (mg/dL)	6.2 (1.2)		5.2 (1.1)		< 0.01	5.8 (1.1)		4.6 (1.0)		< 0.01
Organ damage (yes)										
Cardiovascular disease	71 (7.5)		18 (4.5)		0.03	78 (8.1)		31 (3.3)		< 0.01
Cerebrovascular disease	18 (1.9)		8 (2.0)		0.88	22 (2.3)		29 (3.1)		0.29
CKD (eGFR < 60 mL/min/1.73 m^2^)	192 (20.2)		55 (13.9)		0.01	210 (21.7)		135 (14.2)		< 0.01
eGFR (mL/min/1.73 m^2^)	72.0 (15.8)		74.2 (14.9)		0.02	71.0 (14.9)		75.3 (15.4)		< 0.01
Liver function										
Aspartate aminotransferase (U/L)	27.2 (13.1)		24.8 (11.4)		< 0.01	22.5 (7.2)		22.0 (6.2)		0.14
Alanine aminotransferase (U/L)	33.1 (22.9)		25.9 (19.3)		< 0.01	20.2 (9.8)		16.8 (7.2)		< 0.01

SD, standard deviation; N, number; SBP, systolic blood pressure; DBP, diastolic blood pressure; CKD, chronic kidney disease; eGFR, estimated glomerular filtration rate; MASLD, metabolic dysfunction-associated steatotic liver disease

Table 3 summarizes the number of participants who met each diagnostic criterion for MASLD by sex. Fatty liver, diagnosed by CT scan and abdominal ultrasonography, was identified in 967 (50.4%) men and 405 (30.1%) women. Table 3 also presents the number of associated cardiometabolic risk factors [8] and SUA levels, the primary factor of interest in this study.

**Table 3:** Sex-specific distribution of diagnostic criteria for MASLD

	Mean (SD) or *N* (%)				*P*
	Men (*n* = 1,917)		Women (*n* = 1,347)		
Steatotic liver disease (present)	967 (50.4)		405 (30.1)		< 0.01
By computed tomography scan	584 (30.4)		204 (15.1)		< 0.01
By ultrasound examination	932 (48.6)		397 (29.5)		< 0.01
Obesity-related items					
Body mass index (kg/m^2^) ≥ 23 kg/m^2^	1,244 (64.9)		441 (32.7)		< 0.01
Waist circumference (cm) ≥ 94 cm (M) ≥ 80 cm (W)	363 (18.9)		583 (43.3)		< 0.01
Glucose intolerance-related items					
Fasting blood glucose (mg/dl) ≥ 100 mg/dL	1,073 (55.9)		455 (33.8)		< 0.01
Hemoglobin A1c (%) ≥ 5.7%	1,167 (60.8)		804 (59.7)		0.50
Type 2 diabetes mellitus (yes)	271 (14.1)		70 (5.2)		< 0.01
Diabetes medication (yes)	263 (13.7)		64 (4.8)		< 0.01
Hypertension-related items					
≥ 130 (SBP) and/or 85 (DBP) (mmHg)	775 (40.4)		474 (35.2)		< 0.01
Hypertension medication (yes)	688 (35.9)		252 (18.7)		< 0.01
Lipid abnormalities-related items					
Plasma triglycerides (mg/dL) ≥ 150 mg/dL	455 (23.7)		139 (10.3)		< 0.01
Plasma high-density lipoprotein cholesterol (mg/dL) ≤ 40 mg/dL (M) ≤ 50 mg/dL (W)	222 (11.6)		150 (11.1)		0.68
Dyslipidemia medication (yes)	541 (28.2)		284 (21.1)		< 0.01
Hyperuricemia-related items					
Uric acid (mg/dL) ≥ 7.0 mg/dL	393 (20.5)		44 (3.3)		< 0.01
Hyperuricemia medication (yes)	308 (16.1)		12 (0.9)		< 0.01

SD, standard deviation; N, number; SBP, systolic blood pressure; DBP, diastolic blood pressure; MASLD, metabolic dysfunction-associated steatotic liver disease

**Fig. 1:**
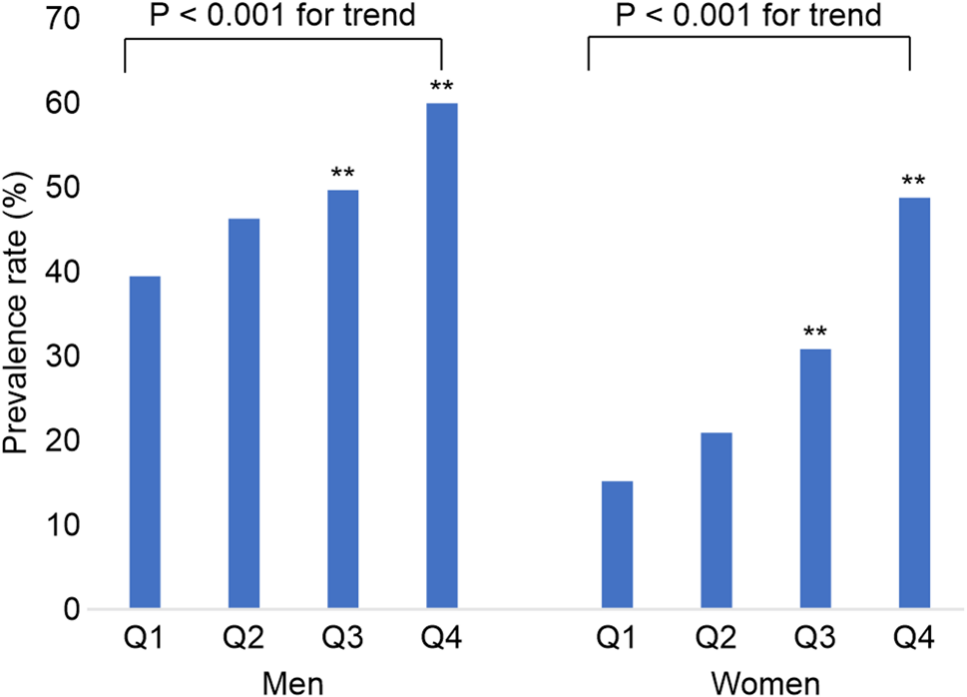
Prevalence of MASLD across SUA quartiles in men and women. P values are shown for each quartile ** *P* < 0.01. MASLD, metabolic dysfunction-associated steatotic liver disease; SUA, serum uric acid

All participants were categorized into quartiles based on SUA levels. The PR% of MASLD across SUA quartiles is illustrated in Fig. 1. The participants in higher SUA quartiles demonstrated significantly higher PR% of MASLD compared with those in lower quartiles.

Using multivariable logistic regression, higher SUA levels were significantly associated with MASLD (Table 4). In Model 3, AORs among men were 1.45 (95% CI: 1.08–1.97) for Q2, 1.43 (95% CI: 1.04–1.95) for Q3, and 2.00 (95% CI: 1.45–2.76) for Q4, compared with Q1. Among women, the corresponding AORs were 1.45 (95% CI: 0.90–2.35), 2.21 (95% CI: 1.39–3.53), and 3.52 (95% CI: 2.22–5.56), respectively. When SUA was analyzed as a continuous variable, significant associations persisted (men: AOR, 1.25; 95% CI, 1.14–1.38; women: AOR, 1.48; 95% CI, 1.27–1.72).

**Table 4:** Association between serum uric acid quartiles and MASLD using multivariable logistic regression

Sex	SUA	Odds ratio (95% confidence interval) /*P* value								
		Model1			Model2			Model3		
		OR	95%CI	P value	OR	95%CI	P value	OR	95%CI	P value
Men	Q1 (< 5.2)	Ref	Ref	Ref	Ref	Ref	Ref	Ref	Ref	Ref
	Q2 ( ≧ 5.2, < 6.0)	1.35	1.04–1.74	0.03	1.40	1.07–1.83	< 0.01	1.45	1.08–1.97	< 0.01
	Q3 ( ≧ 6.0, < 6.7)	1.51	1.16–1.98	0.01	1.66	1.25–2.20	< 0.01	1.43	1.04–1.95	<0.01
	Q4 ( ≧ 6.7)	2.27	1.74–2.94	< 0.01	2.66	2.00–3.55	< 0.01	2.00	1.45–2.76	<0.01
	As a continuous value	1.33	1.23–1.44	< 0.01	1.41	1.29–1.54	< 0.01	1.25	1.14–1.38	<0.01
Women	Q1 (< 4.1)	Ref	Ref	Ref	Ref	Ref	Ref	Ref	Ref	Ref
	Q2 ( ≧ 4.1, < 4.7)	1.49	1.00–2.23	0.05	1.56	1.04–2.35	0.03	1.45	0.90–2.35	0.13
	Q3 ( ≧ 4.7, < 5.4)	2.42	1.65–3.55	< 0.01	2.68	1.79–4.00	< 0.01	2.21	1.39–3.53	<0.01
	Q4 ( ≧ 5.4)	5.21	3.60–7.53	< 0.01	5.98	4.02–8.91	< 0.01	3.52	2.22–5.56	<0.01
	As a continuous value	1.72	1.53–1.94	< 0.01	1.84	1.62–2.09	< 0.01	1.48	1.27–1.72	<0.01

Adjusted odds ratios (ORs) and 95% confidence intervals (CIs) for MASLD across sex-specific quartiles of serum uric acid (SUA). Model 1 adjusts for age. Model 2 adjusts for age, alcohol consumption (daily drinker), smoking behavior (current smoker), estimated glomerular filtration rate (eGFR), and use of urate-lowering agents. Model 3 additionally adjusts for body mass index (BMI). Quartile 1 (Q1) serves as the reference category. SUA was also analyzed as a continuous variable (per 1.0 mg/dL increase). *p* < 0.05 was considered statistically significant

Since MASLD prevalence exceeded 40%, we additionally estimated PRs using Poisson regression with robust variance (Table 5). The overall pattern of associations in the Poisson models closely paralleled the logistic regression results; however, the effect sizes were attenuated, as expected under the high outcome prevalence.

**Table 5:** Prevalence ratios for MASLD across serum uric acid quartiles using Poisson regression with robust variance

Sex	SUA quartile	Model 1 PR (95%CI)	*p*	Model 2 PR (95%CI)	*p*	Model 3 PR (95%CI)	*p*
Men	Q1 (< 5.2)	1.00	—	1.00	—	1.00	—
	Q2 ( ≧ 5.2, < 6.0)	1.18 (1.02–1.37)	0.03	1.19 (1.03–1.38)	0.02	1.14 (0.99–1.31)	0.06
	Q3 ( ≧ 6.0, < 6.7)	1.26 (1.09–1.46)	< 0.01	1.31 (1.12–1.52)	< 0.01	1.19 (1.03–1.37)	0.02
	Q4 ( ≧ 6.7)	1.50 (1.31–1.72)	< 0.01	1.60 (1.39–1.84)	< 0.01	1.40 (1.22–1.60)	< 0.01
Women	Q1 (< 4.1)	1.00	—	1.00		1.00	—
	Q2 ( ≧ 4.1, < 4.7)	1.38 (1.00–1.91)	0.05	1.43 (1.03–1.99)*	0.04*	1.14 (0.83–1.56)*	0.41*
	Q3 ( ≧ 4.7, < 5.4)	1.97 (1.46–2.66)	< 0.01	2.10 (1.53–2.89)	< 0.01	1.63 (1.20–2.22)	< 0.01
	Q4 ( ≧ 5.4)	3.15 (2.38–4.18)	< 0.01	3.39 (2.54–4.54)	< 0.01	2.18 (1.63–2.92)	< 0.01

Prevalence ratios (PRs) and 95% confidence intervals estimated using Poisson regression with robust variance. Model definitions are identical to those in Table 4, except that PRs were estimated instead of odds ratios. *For women in SUA quartile 2, urate-lowering agents were excluded from Models 2 and 3 due to complete separation (only one user, none with MASLD). *p* < 0.05 was considered statistically significant

**Fig. 2:**
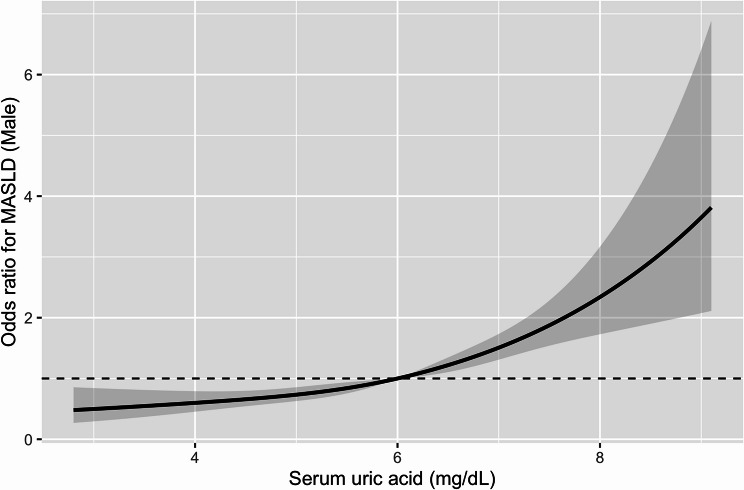
Dose–response association between serum uric acid and MASLD in men. Restricted cubic spline (RCS) curve showing the dose–response association between serum uric acid (SUA) levels and MASLD among men. Odds ratios (solid line) and 95% confidence intervals (shaded area) were estimated from multivariable logistic regression models adjusted for age, alcohol consumption, smoking status, estimated glomerular filtration rate (eGFR), and use of uric acid-lowering medication. The reference value was set at an odds ratio of 1.0 (dashed line). Four knots were used for spline modeling

**Fig. 3:**
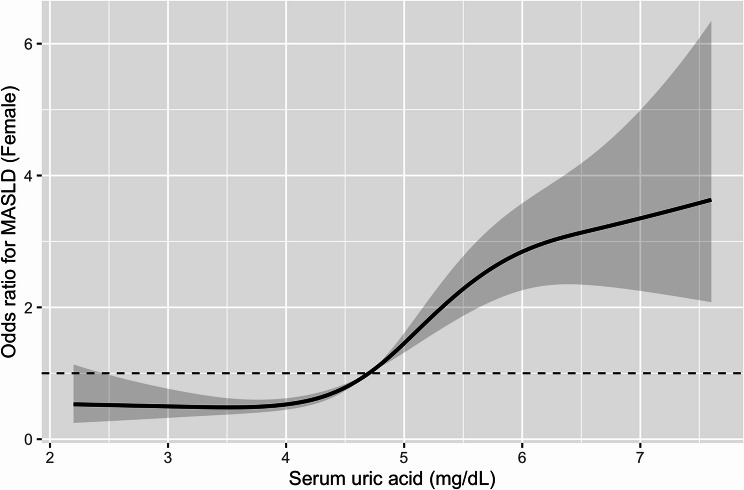
Dose–response association between serum uric acid and MASLD in women. Restricted cubic spline (RCS) curve illustrating the dose–response association between serum uric acid (SUA) levels and MASLD among women. Odds ratios (solid line) and 95% confidence intervals (shaded area) were derived from multivariable logistic regression models adjusted for age, alcohol consumption, smoking status, estimated glomerular filtration rate (eGFR), and use of uric acid-lowering medication. The reference value was defined as an odds ratio of 1.0 (dashed line). Four knots were applied for spline construction

In Model 3, higher SUA quartiles were associated with a higher prevalence of MASLD in both men and women. Among men, PRs increased across SUA quartiles, reaching the highest value in Q4 compared with Q1. Similarly, among women, PRs showed a stronger graded association, with markedly elevated MASLD prevalence in the highest SUA quartiles. Dose–response analyses using restricted cubic spline models demonstrated a positive association between serum uric acid (SUA) levels and MASLD in both sexes (Figs. 2 and 3). In men, the overall association between SUA and MASLD was statistically significant (*P* < 0.01), whereas no significant evidence of non-linearity was observed (P for non-linearity = 0.31). In contrast, SUA showed a significant overall association with MASLD (*P* < 0.01) as well as a statistically significant non-linear relationship in women (P for non-linearity < 0.01).

To address potential circularity arising from MASLD being defined by metabolic components correlated with SUA, we performed a sensitivity analysis restricted to imaging-defined hepatic steatosis regardless of metabolic criteria (Supplementary Tables S1–S3, Additional file 1). In both men and women, higher SUA quartiles were consistently associated with a greater prevalence of imaging-defined steatosis (diagnosed by either CT or ultrasonography), as presented in Supplementary Tables S1 and S2; these associations remained significant in fully adjusted models.

In the ultrasound-only subgroup (Supplementary Table S3), although the effect sizes were smaller and most associations were not statistically significant, the direction of association was similar to the main analysis. CT-only analyses could not be performed due to the small number of CT-defined steatosis cases.

E-values of the fully adjusted Model 3 estimates indicated that an unmeasured confounder would require a substantial association with both SUA and MASLD to fully explain the observed results (Supplementary Table S4, Additional file 1). Dose–response curves generated using restricted cubic spline logistic models showed a monotonic increase in MASLD prevalence with rising SUA levels in both sexes (Figs. 2 and 3).

## Discussion

This study demonstrated that MASLD was significantly associated with SUA levels in both men and women. The relationship between SUA quartiles and MASLD prevalence remained significant after adjustment for potential confounding factors. Although many previous studies have assessed the relationship between SUA and NAFLD, scarce data are available under the updated MASLD definition, which incorporates metabolic dysfunction into the disease framework. In this context, our findings might add to the emerging MASLD-specific evidence base.

The present results showed a significant association between MASLD prevalence and SUA levels, which are consistent with those in previous reports. Several studies have reported similar associations between MASLD and SUA levels [16, 22]. For instance, a study conducted in a Chinese adult population of 3,829 individuals aged 18 years or older, who were diagnosed with fatty liver by ultrasonography and assessed for hyperuricemia, classified SUA into low, medium, and high levels. Among these participants, 1,737 were diagnosed with MASLD. Mean SUA levels were higher in participants with MASLD (5.79 ± 1.50 mg/dL) compared with those without MASLD (5.03 ± 1.35 mg/dL). Furthermore, for every 1 mg/dL increase in SUA, the risk of MASLD increased by 14% (odds ratio [OR] = 1.14, *p* = 0.0004) [21]. Other relevant studies have been conducted in Japanese populations. One cross-sectional analysis involving 58,110 participants assessed the complication rate of MASLD and metabolic and alcohol-related liver disease (MetALD) by allocating participants into quartile groups based on SUA levels. The results showed that higher SUA values were associated with higher complication rates of MASLD/MetALD in both men and women. Additionally, a Cox proportional hazards model was applied to evaluate the relationship between baseline SUA levels and incident MASLD/MetALD during the follow-up period among 22,364 participants without MASLD/MetALD at baseline. The findings demonstrated that participants with higher SUA levels had higher incidence rates of MASLD/MetALD in both sexes [18]. Together with our findings, these results support the relevance of SUA as a metabolic indicator associated with MASLD within the framework of the updated disease definition. Furthermore, the present study contributes to additional evidence from a Japanese cohort, in which MASLD-specific analyses remain relatively limited.

Furthermore, using multivariate analysis in this study, the OR outcome for MASLD significantly increased even when SUA levels were below the conventional hyperuricemia threshold of 7.0 mg/dL. The risk was elevated when uric acid levels exceeded 6.0 mg/dL in men and 4.1 mg/dL in women, suggesting that even moderate uric acid levels might be clinically relevant in MASLD risk. Notably, our study is among the first to demonstrate, in a large Japanese cohort, that women are at increased risk of MASLD even at SUA levels below the conventional definition of hyperuricemia, highlighting a sex-specific vulnerability that has been underrecognized. In addition, restricted cubic spline analyses provided further insights into the shape of the association between SUA and MASLD. While the SUA–MASLD relationship appeared approximately linear in men, women exhibited a clear non-linear pattern, characterized by a relatively flat risk at lower SUA levels followed by a steeper increase beyond mid-range concentrations. This finding suggests that women may be particularly susceptible to increases in SUA even at levels below the conventional hyperuricemia threshold.

Sex-specific differences in the association between SUA and MASLD observed in this study might be partly explained by physiological and metabolic mechanisms. Women generally have lower baseline SUA levels than do men because estrogen has uricosuric effects that enhance renal uric acid excretion. After menopause, this protective effect diminishes and SUA levels tend to rise, potentially amplifying the metabolic impact of elevated SUA [22]. In addition, epidemiological evidence from Japanese general populations indicates that SUA is more strongly associated with metabolic syndrome and its components in women than in men, even within relatively low SUA ranges [23]. These observations support the possibility that women may be more metabolically sensitive to SUA elevations, which might contribute to the steeper SUA–MASLD gradient seen in our cohort.

Several mechanisms might explain the association. One key factor is obesity. Excessive accumulation of visceral fat induces insulin resistance, and hyperinsulinemia reduces renal uric acid excretion. Additionally, insulin resistance activates XOR, an enzyme that promotes uric acid production; thereby, contributing to hyperuricemia. Given the close relationship between obesity and fatty liver, numerous studies have reported associations between NAFLD and uric acid. Although MASLD and NAFLD are differently defined, they share substantial similarities in metabolic disease associations. These parallels suggest that uric acid might act as a risk factor for MASLD [24].

Furthermore, since renal function and the use of urate-lowering therapy substantially affect SUA concentrations, we reconstructed the multivariable models to include eGFR, urate-lowering medication use, and BMI as additional covariates. The positive association between SUA and MASLD remained consistent across all models, indicating that the findings were robust even after careful adjustment for these potential confounders. Additional analyses using Poisson regression and E-values yielded results consistent with the primary findings, further supporting the robustness of the observed associations.

This study has some limitations. First, selection bias might have influenced the results because participants were limited to individuals who traveled to a university hospital in Tokyo for medical examinations. These participants might have been more health-conscious compared with residents in rural areas. Expanding future research to include more diverse cohorts would help address this concern. Second, some essential data were not collected, including detailed information on prescribed medications for hypertension, DM, dyslipidemia, and hyperuricemia. In addition, the dataset did not include sufficient etiologic or metabolic information to classify newer steatotic liver disease subcategories such as MetALD or cryptogenic steatotic liver disease; therefore, subgroup analyses based on these entities could not be performed. Insulin resistance, endothelial dysfunction, oxidative stress, and inflammatory status were also not assessed. In addition, although our models adjusted for major confounders including BMI, we did not incorporate metabolic biomarkers such as lipid profiles or glucose parameters because these variables overlap with MASLD diagnostic criteria and could introduce overadjustment. Future causal-inference-oriented studies may more comprehensively evaluate optimal covariate structures. Third, some lifestyle-related variables lacked precision, as the information was derived from self-administered questionnaires, which might have introduced misclassification. Fourth, since microbiome-related data were unavailable, we could not evaluate the potential contribution of gut dysbiosis to SUA or MASLD. Given emerging evidence linking the gut microbiome to uric acid metabolism, future studies integrating microbiome assessments are warranted. Fifth, detailed dietary information, including fructose-containing food and beverage intake, was not available. Since fructose consumption could influence SUA levels, the lack of dietary data limits our ability to account for this potential source of variation. Future studies incorporating comprehensive dietary assessments are needed for clarifying these relationships. Finally, the cross-sectional design limited the ability to infer causal relationships between elevated SUA and MASLD.

Collectively, these limitations underscore the importance of future prospective studies with larger and more diverse populations, comprehensive clinical and lifestyle data, and validated measurement tools to clarify the causal role of SUA in MASLD pathogenesis.

## Conclusions

This study demonstrated that elevated SUA levels were significantly associated with the prevalence of MASLD in both men and women, and these associations remained robust after multivariable adjustment including BMI, renal function, lifestyle factors, and use of urate-lowering medication. Notably, women showed an increased risk of MASLD even at SUA levels below the conventional hyperuricemia threshold (7.0 mg/dL), with evidence of a non-linear risk increase, particularly at SUA levels below the conventional hyperuricemia threshold, suggesting a sex-specific metabolic sensitivity to SUA. Sensitivity analyses restricted to imaging-defined steatosis further supported the consistency of these findings.

Collectively, these results highlight SUA as a clinically relevant metabolic marker linked to MASLD and underscore the importance of incorporating sex-specific risk assessment into MASLD prevention and management strategies. Further prospective studies are warranted to clarify causal pathways.

## Supplementary Information

Below is the link to the electronic supplementary material.

Supplementary Material 1

## Data Availability

The datasets generated and/or analyzed during the current study are not publicly available due to privacy and ethical considerations but are available from the corresponding author on reasonable request. Data supporting the conclusions of this paper are included within the article.

## Abbreviations

ANOVA: Analysis of variance
MASLD: Metabolic dysfunction-associated steatotic liver disease
MetALD: Metabolic and alcohol-related liver disease
NAFLD: Non-alcoholic fatty liver disease
SUA: Serum uric acid
T2DM: Type 2 diabetes mellitus
BMI: Body mass index
WC: Waist circumference
PRs: Prevalence ratios
SBP: Systolic blood pressure
DBP: Diastolic blood pressure
FPG: Fasting plasma glucose
HbA1c: Hemoglobin A1c
TC: Total cholesterol
LDL-C: Low-density lipoprotein cholesterol
HDL-C: High-density lipoprotein cholesterol
TGs: Triglycerides
AST: Aspartate aminotransferase
ALT: Alanine aminotransferase
DM: Diabetes mellitus
OR: Odds ratio
AOR: Adjusted odds ratio
CI: Confidence interval
