# Supplementary material for: Positive association between serum uric acid and metabolic dysfunction-associated steatotic liver disease: insights from a Japanese health checkup cohort
Source: BMC Endocr Disord. 2026 Jan 30;26:72. doi: 10.1186/s12902-026-02174-5 (PMC12930785; doi:10.1186/s12902-026-02174-5)
Supplement: Supplementary file 1 — Supplementary Material 1 [file 12902_2026_2174_MOESM1_ESM.docx]

**Supplementary Table S1. Logistic regression analysis for imaging-defined hepatic steatosis according to serum uric acid quartiles**

| Sex | SUA | Odds ratio (95% confidence interval) /P value | | | | | | | | |
| --- | --- | --- | --- | --- | --- | --- | --- | --- | --- | --- |
|  |  | Model1 | | | Model2 | | | Model3 | | |
|  |  | OR | 95%CI | P value | OR | 95%CI | P value | OR | 95%CI | P value |
| Men | Q1 (< 5.2) | Ref | Ref | Ref | Ref | Ref | Ref | Ref | Ref | Ref |
|  | Q2 (≧ 5.2, < 6.0) | 1.31 | 1.01–1.70 | 0.04 | 1.36 | 1.05–1.78 | 0.02 | 1.39 | 1.03–1.88 | 0.03 |
|  | Q3 (≧ 6.0, < 6.7) | 1.50 | 1.15–1.96 | <0.01 | 1.64 | 1.24–2.17 | <0.01 | 1.41 | 1.03–1.92 | 0.03 |
|  | Q4 (≧ 6.7) | 2.25 | 1.73–2.92 | <0.01 | 2.63 | 1.97–3.51 | <0.01 | 1.97 | 1.43–2.72 | <0.01 |
|  | As a continuous value | 1.33 | 1.23–1.44 | <0.01 | 1.41 | 1.29–1.53 | <0.01 | 1.24 | 1.13–1.37 | <0.01 |
| Women | Q1 (< 4.1) | Ref | Ref | Ref | Ref | Ref | Ref | Ref | Ref | Ref |
|  | Q2 (≧ 4.1, < 4.7) | 1.44 | 0.97–2.13 | 0.07 | 1.50 | 1.00–2.24 | 0.05 | 1.39 | 0.88–2.22 | 0.16 |
|  | Q3 (≧ 4.7, < 5.4) | 2.29 | 1.57–3.35 | <0.01 | 2.57 | 1.73–3.83 | <0.01 | 2.11 | 1.34–3.35 | <0.01 |
|  | Q4 (≧ 5.4) | 4.78 | 3.33–6.84 | <0.01 | 5.55 | 3.76–8.20 | <0.01 | 3.30 | 2.11–5.16 | <0.01 |
|  | As a continuous value | 1.70 | 1.51–1.92 | <0.01 | 1.82 | 1.60–2.06 | <0.01 | 1.46 | 1.26–1.70 | <0.01 |

Odds ratios (ORs) and 95% CIs for hepatic steatosis (fatty liver vs. non-fatty liver) based on ultrasound or CT findings. Models were adjusted identically to Table 4. Model 3 includes BMI. This analysis was performed to evaluate the association independent of MASLD diagnostic criteria (metabolic components). *p* <0.05 was considered statistically significant.

**Supplementary Table S2. Prevalence ratios for hepatic steatosis using Poisson regression with robust variance**

| **Sex** | **SUA quartile** | **Model 1 PR (95%CI)** | **p** | **Model 2 PR (95%CI)** | **p** | **Model 3 PR (95%CI)** | **p** |
| --- | --- | --- | --- | --- | --- | --- | --- |
| **Men** | Q1 (< 5.2) | 1.00 | — | 1.00 | — | 1.00 | — |
|  | Q2 (≧ 5.2, < 6.0) | 1.16 (1.01–1.35) | 0.04 | 1.17 (1.02–1.35) | 0.03 | 1.12 (0.98–1.29) | 0.09 |
|  | Q3 (≧ 6.0, < 6.7) | 1.25 (1.08–1.44) | 0.03 | 1.29 (1.12–1.50) | <0.01 | 1.18 (1.03–1.36) | 0.02 |
|  | Q4 (≧ 6.7) | 1.49 (1.30–1.70) | <0.01 | 1.58 (1.37–1.81) | <0.01 | 1.38 (1.21–1.59) | <0.01 |
| **Women** | Q1 (< 4.1) | 1.00 | — | 1.00 |  | 1.00 | — |
|  | Q2 (≧ 4.1, < 4.7) | 1.34 (0.98–1.83) | 0.07 | 1.38 (1.00–1.90)^＊^ | 0.05^＊^ | 1.13 (0.83–1.53)^＊^ | 0.44^＊^ |
|  | Q3 (≧ 4.7, < 5.4) | 1.88 (1.40–2.53) | <0.01 | 2.03 (1.49–2.75) | <0.01 | 1.58 (1.17–2.13) | <0.01 |
|  | Q4 (≧ 5.4) | 2.91 (2.23–3.80) | <0.01 | 3.15 (2.40–4.15) | <0.01 | 2.05 (1.56–2.71) | <0.01 |

PRs and 95% CIs for fatty liver vs. non-fatty liver. Model 1 adjusts for age. Model 2 adjusts for age, alcohol consumption, smoking status, eGFR, and urate-lowering therapy. Model 3 additionally adjusts for BMI. ^*^For women in Q2, urate-lowering therapy was excluded from Models 2 and 3 due to complete separation. *p* <0.05 indicates statistical significance.

**Supplementary Table S3. Sensitivity analysis restricted to participants assessed by abdominal ultrasonography**

| **Sex** | **SUA quartile** | **Model 1 PR (95%CI)** | **p** | **Model 2 PR (95%CI)** | **p** | **Model 3 PR (95%CI)** | **p** |
| --- | --- | --- | --- | --- | --- | --- | --- |
| **Men** | Q1 (< 5.2) | 1.00 | — | 1.00 | — | 1.00 | — |
|  | Q2 (≧ 5.2, < 6.0) | 1.01 (0.97–1.05) | 0.53 | 1.01 (0.98–1.06) | 0.48 | 1.03 (0.99–1.07) | 0.20 |
|  | Q3 (≧ 6.0, < 6.7) | 1.03 (0.99–1.07) | 0.16 | 1.03 (0.99–1.07) | 0.11 | 1.03 (0.99–1.07) | 0.12 |
|  | Q4 (≧ 6.7) | 1.03 (0.99–1.07) | 0.11 | 1.03 (0.99–1.08) | 0.09 | 1.03 (0.99–1.07) | 0.15 |
| **Women** | Q1 (< 4.1) | 1.00 | — | 1.00 |  | 1.00 | — |
|  | Q2 (≧ 4.1, < 4.7) | 1.04 (0.96–1.13) | 0.35 | 1.02 (0.96–1.09)^＊^ | 0.50^＊^ | 1.01 (0.95–1.08)^＊^ | 0.71^＊^ |
|  | Q3 (≧ 4.7, < 5.4) | 1.05 (0.98–1.13) | 0.15 | 1.04 (0.99–1.10) | 0.15 | 1.04 (0.98–1.09) | 0.18 |
|  | Q4 (≧ 5.4) | 1.05 (0.98–1.13) | 0.15 | 1.04 (0.98–1.10) | 0.18 | 1.03 (0.97–1.09) | 0.31 |

PRs and 95% CIs for MASLD using Poisson regression restricted to participants evaluated by ultrasound only. CT-derived steatosis cases were excluded due to small case numbers and unstable estimates. Model adjustment structure is identical to Table 5. This analysis evaluates robustness across imaging modalities. ^*^ For women in SUA quartile 2, Models 2 and 3 were estimated using negative binomial regression with robust variance because Poisson regression did not converge due to numerical instability related to the distribution of eGFR. *p* <0.05 was considered significant.

**Supplementary Table S4. E-values for the association between serum uric acid quartiles (Q4 vs Q1) and MASLD in fully adjusted models**

| Sex | PR (Q4 vs Q1) | 95% CI (Lower) | E-value (Point estimate) | E-value (Lower CI) |
| --- | --- | --- | --- | --- |
| Men | 1.40 | 1.22 | 2.15 | 1.74 |
| Women | 2.18 | 1.63 | 3.78 | 2.64 |

E-values quantify the minimum strength of association that an unmeasured confounder would need to fully explain away the observed association between SUA and MASLD. Values were calculated from Model 3 prevalence ratios.
